# CTAD taskforce: genetic therapies in Alzheimer’s disease

**DOI:** 10.1016/j.tjpad.2025.100269

**Published:** 2025-07-09

**Authors:** D. Jakabek, A.M. Isaacs, B. De Strooper, M. Tuszynski, R. Lane, O. Uspenskaya, E. McDade, M.S. Rafii, C.J. Mummery

**Affiliations:** a University College London, London, UK; b UKDRI@ University College London, London, UK and VIB@KULeuven, Belgium; c University of California San Diego, San Diego, USA; d IONIS Pharmaceuticals, Carlsbad, USA; e Prevail Therapeutics, a wholly-owned subsidiary of Eli Lilly and Company, USA; f Washington University in St. Louis, Saint Louis, USA; g University of Southern California, Los Angeles, USA

**Keywords:** RCTs, Genetic therapy, siRNA, ASO, AAV

## Abstract

There are an increasing number of genetic approaches to treating Alzheimer’s disease and other dementias, with some promising results from early-phase trials. This prompted the convention of the first EU-US CTAD Task Force on genetic therapies in Alzheimer’s disease in October 2024. Preclinical studies and clinical trials of genetic therapies in Alzheimer’s disease and other dementias are presented here with key lessons for the field. Importantly, there are several challenges and opportunities unique to neurogenetic therapies which were reviewed and discussed, including means of genetic manipulation, adverse events, monitoring, timing of therapy, and the importance of patient involvement in trial design. Continued collaboration across disciplines will accelerate development of neurogenetic therapeutics.

## 1 Introduction

Genetic therapies hold promise to address the fundamental underlying causes of neurodegenerative disease. After a long and often disappointing path in clinical trials aiming to treat Alzheimer’s disease and related dementias, in recent years, the approval of the first disease modifying therapies in the form of anti-amyloid monoclonal antibodies (mABs) has re-energised the academic community. Anti-amyloid mABs have modest effects in slowing cognitive decline in mild clinical Alzheimer’s disease (AD) but do not halt the disease progress, and evidence suggests that their benefit is greater earlier in the course of the disease [1]. In contrast to monoclonal antibodies that bind to and facilitate the clearance of protein from the brain, genetic therapies modify underlying protein synthesis, working upstream of the deposition of abnormal proteins. In doing so they may have a greater benefit - substantially delaying, or even preventing, disease onset and/or progression.

Genetic therapies could delay or prevent the onset and progression of preclinical and clinical neurodegenerative diseases. As a broad term, genetic therapies encompass any methods modifying genes or their expression, whereas gene therapies target genes directly. RNA-based or gene silencing therapies can reduce the production of proteins such as amyloid or tau by altering the level of messenger RNA (mRNA) from which the protein is translated. Depending on the mechanism of action and chemical modifications, effects may last from months to a year or more [2,3]. In contrast, gene therapies aim to permanently alter DNA, and thereby potentially offering “one and done” treatments whereby a single treatment may have permanent effects in modifying the course of disease. Both of these therapeutic modalities could offer savings in patient treatment burden and subsequent health economics.

The conduct of several genetic therapy clinical trials for neurodegenerative disease prompted the formation of the first International Clinical Trials in Alzheimer's Disease Task Force for Genetic Therapies in Alzheimer’s Disease at the 2024 Clinical Trials in Alzheimer’s Disease (CTAD) meeting. The Task Force consisted of a broad range of representatives across basic sciences and clinicians, in both academic and industry roles, to consider past and upcoming neurogenetic therapies. This paper will outline genetic therapy clinical trials in Alzheimer’s disease and frontotemporal dementia, as well as relevant nonclinical studies to highlight the broad spectrum of neurogenetic therapies being explored. Next, we specifically consider lessons learnt from past trials, both with negative and positive results, and consider the challenges in forthcoming trials for neurogenetic therapies.

## 2 Methods of genetic therapy in neurodegenerative disease

Neurogenetic therapies via RNA- or DNA-based mechanisms broadly fall into three different approaches: the expression of functional genes may be [1] reduced or [2] increased, or genes or mRNA may be edited or alternatively spliced to [3] produce an altered protein (for detailed review see [4]). Each aim can be achieved using different delivery or modification mechanisms and will be briefly outlined below. Examples are provided of recent AD and other dementia drug trials in Table 1 with further details later in the manuscript.

**Table 1:** Recent trials of genetic therapies for Alzheimer's disease and other dementias.

	Target and mechanism	Condition	Trial number	Phase
IONIS-MAPT_Rx_ / BIIB080	MAPT ASO	AD	NCT03186989 NCT05399888	Phase 1b Phase 2
NIO752	MAPT ASO	AD	NCT06372821 NCT05469360	Phase 1b Phase 1b
ION269	APP ASO	Down Syndrome at risk of AD	NCT06673069	Phase 1b
ION464	Alpha-synuclein (SNCA) ASO	Multiple systems atrophy	NCT04165486	Phase 1
LY3954068	MAPT siRNA	AD	NCT06297590	Phase 1
mivelserin	APP siRNA	Early-onset AD	NCT05231785	Phase 1
mivelserin	APP siRNA	CAA	NCT06393712	Phase 2
CERE-110	*NGF* gene via AAV2	AD	NCT00087789	Phase 2
AAV2-BDNF	*BDNF* gene delivery via AAV2	AD	NCT05040217	Phase 1
LX1001	*APOE2* gene delivery via AAVrh.10	AD with APOE homozygotes	NCT03634007	Phase 1/2
WVE-004	C9orf72 ASO	C9orf72-associated frontotemporal dementia or amyotrophic lateral sclerosis	NCT04931862	Phase 1b/2a
PR006	*GRN* gene delivery via recombinant AAV9	Frontotemporal dementia with progranulin mutation	NCT04408625	Phase 1/2
AVB-101	*GRN* gene delivery via AAV9	Frontotemporal dementia with progranulin mutation	NCT06064890	Phase 1/2
PBFT02	*GRN* gene delivery via AAV1	Frontotemporal dementia with progranulin or c9orf72 mutation	NCT04747431	Phase 1/2

### 2.1 Gene silencing approaches to reduce expression

**Fig. 1:**
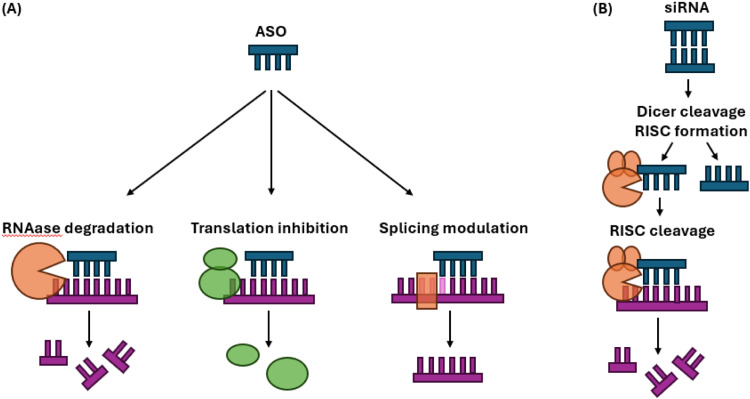
Schematic of (A) ASO and (B) siRNA mechanisms of action. ASOs can either target mRNA for RNAase-mediated degradation, block translation of mRNA by ribosomes, or modulate splicing to alter mRNA. Double-stranded siRNA requires cleavage into single strands by dicer enzyme, before binding to RNA-induced silencing complex (RISC) which cleaves target mRNA.

Gene silencing aims to reduce the levels of gene-encoded mRNAs. This can be achieved through directly targeting the gene’s DNA using DNA-targeting CRISPR-Cas systems, or targeting mRNAs for degradation using RNA-targeting CRISPR-Cas systems. More commonly, it is achieved through RNA-based medicines that are synthetic nucleic acid sequences that include antisense oligonucleotides (ASO), small interfering RNA (siRNA), or microRNA (miRNA). ASOs bind to mRNA/pre-mRNA in the nucleus and either recruit an enzyme to degrade target mRNA, inhibit translation, or alter the splicing of pre-mRNA (Fig. 1A). siRNA/miRNA also make use of a catalytic process, but within the cytoplasm, guiding an RNA-induced silencing complex to cleave mRNA (Fig. 1B). The durability of these therapies is long, increasing from monthly or every few months to every 6 to 12 months. Despite this durability, effects are dose-dependent and fully reversible. Importantly, RNA-based therapeutics are effective regardless of the conformations and locations of toxic proteins because both methods work to degrade mRNA that is transcribed from DNA, from which protein is translated,. This is in contrast to anti-amyloid mABs which may only bind to specific forms of extracellular monomeric, oligomeric, and/or fibrillar forms of amyloid-beta. RNA-based therapeutics can potentially, therefore, be more effective for intracellular proteins such as tau and alpha-synuclein. As ASOs and siRNA are larger than small molecules, they cannot cross the blood brain barrier (BBB), so administration is by lumbar intrathecal bolus injection in current trials.

Trials in AD and other dementias reduce gene expression using either ASO or siRNA methods (Table 1). Tofersen (Qalsody), an ASO targeting *SOD1* mRNA, has received FDA accelerated approval for amyotrophic lateral sclerosis due to *SOD1* mutation on the basis of its ability to in amyotrophic lateral sclerosis reduce neurofilament light chain in blood and CSF and SOD1 protein patients with SOD1 ALS; and confirmation for full approval in a post-marketing trial is ongoing [5].

### 2.2 Gene product supplementation or replacement

Gene supplementation aims to restore healthy genetic code or functional mRNA for normal protein translation. This can occur directly via introduction of functional or missing genes to the nucleus via viral vectors, most commonly adeno-associated virus (AAV). Within AD, this approach has been used to supplement nerve growth factor (NGF; [6,7]) and brain-derived neurotrophic factor (BDNF), and also to by LX1001 to introduce *APOE2* to *APOE4* homozygotes (see [8] for a more detailed review). This mechanism is also used by PR006 to supplement the *GRN* gene in patients with FTD with *GRN* mutations on one allele [9] and by AVB-101 and PBFT02 (detailed in Table 1). Alternatively, ASOs can achieve the translation of functional protein through modulation of pre-mRNA splicing. This latter approach is taken by nusinersen (Spinraza); the first approved intrathecally administered RNA-based genetic therapy in a neurological disease [10]. In patients with spinal muscular atrophy (SMA), the ASO nusinersen modulates the splicing of exon 7 in the SMN2 pre-mRNA (skipping of this exon in the majority of SMN2 transcripts results in non-functional survival motor protein) leading to the inclusion of exon 7 and increased translation of normal SMN protein from the *SMN2* gene.

### 2.3 Gene editing

Gene editing involves modification of DNA sequences without inducing double strand breaks. CRISPR-Cas9 is the most well-known of these methods, but other methods (e.g. TALENs, zinc finger nucleases) are also available. However, despite advances in the gene editing framework, clinical implementation has been limited due to issues with drug delivery, erroneous off-target editing, immunogenicity, and malignancy [11]. Nonetheless, CRISPR-Cas9 approaches are FDA approved for the treatment of sickle cell disease (exagamglogene autotemcel) and are likely to emerge as important neurodegenerative treatment approaches in the future.

## 3 Current and emerging genetic trials for neurodegenerative disease

### 3.1 Autosomal dominant AD

Autosomal dominant AD (ADAD) is an ideal model for designing genetic therapies for AD, since there are specific pathogenic mutations with relatively clear pathological pathways. ADAD includes patients with duplications or mutations in *PSEN1, PSEN2* and *APP* genes, and individuals with Down Syndrome (DS) that have an additional *APP* gene due to the presence of this gene on their extra chromosome 21. Mutations in *PSEN1* cause alterations in the relative ratios of Aβ species through loss of function of gamma-secretase processing of APP [12]. There are strong correlations between the ratios of longer (≥42) to shorter (<42) Aß lengths and predicted age of onset of ADAD [13,14]. Importantly, mutations for PSEN are predominantly heterozygotic [12]. Thus, there is ongoing preclinical work to find ASO targets for *PSEN1* mutations to inhibit the pathological allele while allowing expression of the healthy allele. Alternatively, in mouse studies with *PSEN1* L435F knock-in variants, the introduction of wild-type human *PSEN1* using AAV restores gamma-secretase activity [15], and progress to human studies is awaited.

### 3.2 Alzheimer’s disease in down syndrome

Down Syndrome is the commonest genetic form of AD, and AD is the leading cause of death in adults with DS over the age of 35 [16,17]. Biomarker profile and sequence in AD from DS are similar to the general population [18] which should enable rapid translation of treatments for ADAD or sporadic AD to the DS population. However, this population has been largely ignored until recently. There is a higher proportion of cerebral amyloid angiopathy in people with DS [19] and anti-amyloid mABs are not yet being prescribed to individuals with DS due to concern for amyloid-related imaging abnormalities and until safety studies are done. Nevertheless, this population is uniquely suited to treatment targets on the amyloid pathway. Since the *APP* gene responsible for amyloid production is located on chromosome 21, people with DS have lifelong overexpression of APP and develop amyloid plaques and tau tangles from about 40 years of age [20,21].

Further evidence of dose-dependent APP pathology is provided by rare individuals with partial trisomy 21 (i.e., with partial triplication of chromosome 21 but without *APP* gene triplication), having normal AD biomarkers in later years [22,23]. The rationale follows that reducing APP expression in people with DS and APP triplication may in turn reduce the risk of developing AD. Accordingly, the HERO trial (NCT06673069) is being conducted in conjunction with the Alzheimer’s Clinical Trials Consortium – Down Syndrome. It is a phase 1b multi-center, open-label, single ascending dose trial assessing safety and tolerability of ION269, an antisense oligonucleotide (ASO) against APP, in adults with DS. Secondary aims include changes in soluble APPalpha and soluble APPbeta. Recruitment has commenced for participants with evidence of amyloid pathology, with study completion estimated in early 2027.

### 3.3 Sporadic AD

The same methodological approach used in ADAD can be applied to sporadic neurodegenerative disease. Although sporadic Alzheimer’s disease lacks a specific single genetic mutation (notwithstanding *APOE4* [24]), the pathophysiological processes of accumulation of amyloid plaques and tau tangles are well described. The genes coding for amyloid and tau, *APP* and *MAPT* respectively, are both targets for RNA-based gene silencing to reduce downstream production of amyloid beta plaques and tau tangles. RNA-based gene silencing reduces production of all forms of protein, both abnormal and normal. Consequently, there may be concern for the consequences of normal protein reduction given known physiological roles for amyloid and tau [25]. Reassuringly, knock out mouse models have shown no clear phenotype in tau and only a mild phenotype in amyloid (e.g., [26,27]); nevertheless, it is critical that we continue to monitor patients treated with these methods to determine any long-term effects of reduction and to understand better what level of reduction is safe.

### 3.4 Amyloid targets

Targeting the amyloid pathway, the ALN-APP-001 trial (NCT05231785) uses mivelsiran, an siRNA for *APP* gene silencing in an early-onset (<65 years) AD cohort via intrathecal injection. It is an ongoing randomized, double-blind, placebo-controlled, Phase 1 single-ascending dose and multiple ascending dose study to evaluate the safety, tolerability, pharmacodynamics and pharmacokinetics of mivelsiran. Interim results after a single intrathecal dose demonstrate a dose-dependent rapid and sustained reduction of soluble APPalpha and APPbeta up to 12 months post-dose, and lower AB40 and AB42 up to 6 months post-dose [3,28]. In this study so far, no treatment related adverse events have been reported. Adverse events were mostly related to lumbar punctures. The final results of the trial are awaited, but this exciting approach has the potential to prevent accumulation of amyloid so potentially could be used in prevention; in addition, there is no removal of amyloid so no risk of ARIA, making it an attractive prospect in patients at higher risk of side effects from anti amyloid immunotherapy such as *APOE4* homozygotes, or patients with DS or cerebral amyloid angiopathy. Indeed, a phase 2 trial of mivelsiran in cerebral amyloid angiopathy is ongoing (NCT06393712).

An alternative approach is to potentially induce known protective pathways to prevent Alzheimer’s disease onset. The Icelandic *APP* mutation is thought to be protective for AD by reducing soluble APPbeta production and subsequent Aß40 and Aß42 reduction [29]. With this knowledge, using AAV delivery of CRISPR-Cas9 gene editing of *APP* in animal models, a sustained shift to reduced toxic soluble APPbeta and reduced Aß plaques was observed [30]. In a similar theme, a small trial of LX1001 supplemented *APOE2* genes to six *APOE4* homozygotic participants with an intraparenchymal delivered AAV vector (NCT03634007). Preliminary results indicate that although APOE levels changed appropriately, downstream changes to amyloid and tau were less consistent [28]. Nevertheless, this highlights the approach of using protective mutations to generate AD genetic therapies and could potentially be applied to other mutations such as the Christchurch *APOE3* mutation [31]. Moreover, this approach provides a possible pathway to “one and done” single dosing to individuals at risk for Alzheimer’s disease.

### 3.5 Tau targets

RNA-based genetic therapies targeting MAPT mRNA in patients with AD may allow for a comprehensive amelioration of all forms of phosphorylated and aggregated tau, regardless of location, as they should reduce the production of all forms of tau protein. With the exception of tau seeds, pathogenic hyperphosphorylated tau is intracellular and less accessible to monoclonal antibodies. Moreover, the toxic species of tau are not well understood. There are many different splice forms, post-translational modifications, and conformations of tau protein. This may explain why multiple anti-tau monoclonal antibodies have been unsuccessful in early stage clinical trials [32]. The uncertainty about which tau species are pathological lends itself to a pragmatic solution: reduce all forms of tau in all compartments. The mRNA transcribed from the *MAPT* gene provides a suitable target for tau protein reduction and this is the approach of the IONIS-MAPT_Rx_/BIIB080 ASO. Lumbar intrathecal bolus administration of this ASO in a phase 1b randomized, placebo-controlled trial (NCT03186989) demonstrated pronounced sustained dose-dependent reductions in CSF total tau, CSF ptau181, and CSF tau/Aβ42 ratios [2]. Similar findings were observed in the long-term extension but also critically, in a subgroup of individuals (those in the last two of four cohorts), there was a clear reduction in levels of tau tangles measured on tau PET [22]. These were small numbers so they need to be validated and confirmed in a larger study; however, this was the first trial demonstrating clear tau tangle reductions [33]. Adverse events related to study drug were mild to moderate, and most were related to lumbar puncture procedures. There were no drug related serious adverse events [2]. A phase 2 trial of IONIS-MAPT_Rx_/BIIB080 in a 76-week randomized, placebo-controlled trial and 96-week open label extension has completed recruitment and is ongoing (CELIA, NCT05399888). There are now several trials targeting the MAPT pathway, including NIO752, an ASO (NCT06372821), and LY3954068, an siRNA (J4T-MC-OLAA trial, NCT06297590). Having had multiple negative anti-tau trials, it is cause for optimism that targeting the mRNA and the production of all tau protein, not particular species of the protein, will result in clear reductions in levels of tau protein. This potential benefit will be both in terms of soluble tau, but critically in terms of a marker of insoluble tau tangle burden in the brain. This is a substantial step forward in our potential ability to change the course of AD and other tauopathies.

### 3.6 AD – trophic pathways

In contrast to reducing toxic gain-of-function in amyloid and tau pathways, enhancing neurotrophic factors are also being explored as a treatment target across different brain regions. Nervous system growth factors prevent neuronal death, activate neuronal function and stimulate synapse formation, all properties that could be useful in treating ongoing neurodegeneration. Initially targeting cholinergic pathways in the basal forebrain, a 2001 small trial in eight patients of intraparenchymally injected fibroblasts expressing nerve growth factor (NGF) slowed cognitive decline and improved FDG PET measures [7]. A follow-up phase 2 multicenter, sham surgery-controlled clinical trial of NGF in AD used adeno-associated virus serotype 2 (AAV2) vectors to directly express NGF in cholinergic regions of the cholinergic basal forebrain [6]. The clinical trial failed to show cognitive benefit, but more than 80 % of AAV2-NGF injections missed their intended targets. Accordingly, new MRI-guided infusion methods were developed. MRI-guided infusions are now being used in a current clinical trial of AAV2-brain derived neurotrophic factor (AAV2-BDNF) gene therapy in patients with mild AD and MCI (NCT05040217). In APP-overexpressing mouse models, BDNF improved memory and rebuilt synapses. In aged and lesioned rats, and aged and lesioned non-human primates, BDNF also improved memory, prevented cell death and activated cell function [34]. Six patients with mild AD have been safely treated to date, and the clinical trial is now enrolling patients with MCI. This clinical program is a good example of the use of viral-mediated therapeutic gene over-expression in an effort to treat AD and other disorders. Growth factor gene therapy is also being tested in clinical trials in Parkinson’s disease (NCT06285643) and ALS (NCT02943850).

### 3.7 Frontotemporal dementia

Frontotemporal dementia has several genetic mutations which can be targeted by genetic therapies. Early phase trials are ongoing for both *GRN* and *C9orf72* mutations.

Mutations in the progranulin gene *GRN* cause reduced levels of functional progranulin via haploinsufficiency and subsequent lysosomal dysfunction, excess neuroinflammation and neurodegeneration [35]. Importantly, heterozygous mutations cause FTLD whereas homozygous mutations cause ultra-rare cases of Neuronal Ceroid Lipofuscinosis type 11 [36] indicating a gene dose-dependent pathology related to *GRN* deficiency. The progranulin gene can be supplemented with PR006, which packages a healthy *GRN* gene within an rAAV9 capsid and is delivered via one-time intracisternal injection. The PROCLAIM trial (NCT04408625) is a phase 1/2 open-label multisite single ascending dose trial to assess safety and effects of PR006 in *GRN* mutation carrier with mild to moderate symptoms (≥1 to ≤15 on CDR plus NACC FTLD sum of boxes). Preliminary results indicate that progranulin levels showed a dose-dependent increase in all participants, sustained at 12 month follow-up [9]. Additionally, the intracisternal administration procedure was well tolerated. CSF pleocytosis was observed in most patients and was asymptomatic, apart from one patient with symptomatic sensorineural hearing loss which was ongoing but recovering at the time of the meeting. CSF pleocytosis was expected and attributed to increased transgene over-expression in dorsal root ganglia leading to an inflammatory response. CSF inflammatory response appeared to be responsive to corticosteroids. Further clinical development is ongoing with expansion of eligibility to earlier symptomatic disease (0.5 to 1 on CDR plus NACC FTLD global score). Additional AAV-based gene therapy approaches to supplement progranulin include the use of intrathalamic AAV9 delivery (NCT06064890) and intracisternal delivery using AAV1 serotype (NCT04747431).

A GGGGCC repeat expansion in *C9orf72* is the most common genetic cause of FTD and of ALS and has also been a target for genetic therapies in clinical trials. Two recent trials were negative on primary endpoints [37]. FOCUS-C9 (NCT04931862) was a phase 1b/2a randomized double-blind placebo controlled trial using intrathecal administration of WVE-004, an ASO targeting *C9orf72* repeat-containing mRNA, in FTD and ALS. Despite target engagement (measured via polyGP using a Simoa immunoassay [38]) in pre-clinical [39] and clinical data, there were no clinical benefits to therapy seen on clinical outcome measures at 24 weeks and the trial was terminated. Similarly, a phase 1 randomized controlled trial of BIIB078 in patients with ALS (NCT03626012), another ASO targeting *C9orf72* repeat-containing mRNA, did not meet primary endpoints [40]. Potential causes include that both trials utilized ASOs targeting sense repeat-derived mRNAs, whereas preclinical work has demonstrated that both sense- and antisense-repeat derived RNA species are present in *C9orf72* FTD/ALS [41] and both are neurotoxic in animal studies [42]. Newer approaches include targeting antisense-repeat derived mRNA using ASOs [43] and AAV-delivered CRISPR-Cas13 has demonstrated target engagement (polyGP as previous) and also reduction in both sense and antisense repeat RNA in animal models and human-derived iPSCs [44], providing a promising alternative methodology to target pathological *C9orf72* mutations.

### 3.8 Lessons, challenges and opportunities

Although genetic therapies for neurodegenerative diseases are in their infancy, there are lessons that can be leveraged from genetic therapies for neurological diseases more broadly. There are general challenges such as optimizing drug delivery, but also specific challenges for class adverse events. Uniquely, patients with neurodegenerative diseases may be asymptomatic for decades, and so the varied challenges and opportunities considered below need to be considered in this context.

### 3.9 Therapy administration

Present delivery of RNA-based therapies involve invasive routes of administration, and there are opportunities to improve both in patient convenience and scalability to the population of patients with dementia. Current genetic therapies do not cross the BBB well and in some cases require administration via intrathecal, intracisternal, intraventricular, or intraparenchymal routes. Side effects from these procedures, such as lower back pain and post-dural puncture headache, are the principal side effects of RNA-based therapy trials to date (e.g., [2,9]). Reassuringly, these adverse effects are usually mild and there is no evidence of trial discontinuation due to administration- related adverse effects.

Current genetic therapy administration methods (particularly those requiring intrathecal or operative administration) are resource expensive but this is countered by the one-off or infrequent dosing requirement, at least in part. Scaling intrathecal delivery can build on existing diagnostic pathways for lumbar punctures, but will require additional consideration of appropriate environment for preparation, safe administration and reliable scheduling. A novel challenge may be faced in service delivery of genetic therapies for dementia, given approved neurogenetic therapies have so far been for rarer conditions with smaller potential patient populations (e.g., SMA). There is likely to be inequity in access at least initially, across different populations, dependent on the resources at the local centers; this will need to be actively monitored and initiatives to support will be important. However, there have been recent advancements in drug delivery such as use of active transport mechanisms that may facilitate simpler administration and better distribution of genetic therapies.

Various methods of delivering RNA-based therapeutics (and other large molecules) across the BBB are in development [45]. Blood-brain barrier disruption via MRI guided focused ultrasound has been explored for improved drug delivery in non-genetic therapies [46], but there are substantial limitations on the scalability of this approach. An alternative, receptor mediated transcytosis can actively transport compounds across the BBB, and is being used via the transferrin receptor for anti-amyloid monoclonal antibodies (e.g., BrainShuttle™ [47,48] and BrainTransporter™ [49]) and for genetic therapies [50]. In preclinical models, transferrin mediated transport can allow ASOs or nanobodies to cross the BBB [51,52]. In addition to improving convenience of delivery, active transport across the BBB also demonstrates more homogenous spread of ASO throughout the CNS compared to intrathecal bolus administration, with reduced CSF spikes in concentration and improved distribution to subcortical structures that is on a par with distribution to hippocampus and cerebral and cerebellar cortices [51]. A further advantage of active transport is that this process may be more scaleable to worldwide populations with dementia.

### 3.10 AAV immunogenicity and antibodies

There is a long list of potential AAV side effects (see [53] for review) but in the context of CNS targeting gene therapies, of particular concern is dorsal root ganglia (DRG) toxicity. This is notable due to being a dose-limiting side effect, since it is thought to be due to transgene over-expression with a subsequent inflammatory response [54]. There is responsiveness to immunosuppression in human studies [9] although less so in animal studies [54]. Nevertheless, DRG toxicity is largely asymptomatic, and it has a monophasic time course. It appears to be agnostic to transgene and AAV vector serotype, but more related to dose level. Thus, dose-finding in trials is needed in establishing the optimal balance between efficacy and toxicity.

Additionally, systemic immune responses, as well as surrogate efficacy biomarker should be monitored since drug inactivation may occur due to pre-existing neutralizing anti-AAV antibodies in gene therapy recipients. AAV antibodies are carried by anywhere from 5 to >50 % of the population with titers varying between AAV serotype, regional location, and gender [55]. For intrathecal and intraparenchymal gene administration, pre-existing antibodies do not appear to be associated with changes in efficacy response [9]. But this is important for intravenous administration, since AAV antibodies may prevent delivery of payload to target tissues, and potentially contribute to excess inflammation leading to adverse events. Thus, additional strategies to allow intravenous AAV administration will be required [56], and there may be restrictions or additional considerations for patient selection for AAV therapies, especially older patients who are more likely to have prior AAV exposure.

### 3.11 CSF pleocytosis

CSF pleocytosis is common to ASO, siRNA, and AAV therapies, but has variable occurrence, and the cause remains unknown. With earlier chemical modifications and less efficient screening, ASO therapy was sometimes associated with marked dose-dependent and time-dependent CSF pleocytosis. For example, dose- and dose-intensity-related CSF pleocytosis was evident in clinical trials for tominersen in patients with Huntington’s disease (HD) [57,58] and also evident, though less so, in the tofersen trial in *SOD1* ALS [59] and in early access programs for ASOs [60]. By contrast, nusinersen is not associated with CSF pleocytosis in children with SMA [61] and only transiently and infrequently in adults [62], although very modest increases in CSF protein and CSF/serum albumin ratio are noted. In AD therapies using AAV, ASO, and siRNA, CSF pleocytosis was noted in LX1001 [18], in one patient receiving IONIS MAPT_Rx_/BIIB080 [2], and no abnormal CSF findings have been seen in interim results following a single dose of mivelsiran [3]. These disparate findings may be due to differing inflammatory potential of antisense molecules, dose levels with higher doses being required for less potent ASOs, dose intensity and differential potential of molecules to accumulate in CNS and meningeal tissue, interaction with more traumatic lumbar puncture procedures, underlying disease-related BBB disruption, or adjuvants causing proinflammatory effects. By contrast in AAV therapy, pleocytosis may be a secondary inflammatory response from DRG toxicity [9]. Reassuringly, across ongoing clinical trials CSF pleocytosis appears to be usually monophasic and asymptomatic. Nevertheless, long-term follow-up is essential to ensure no delayed sequelae.

### 3.12 Combination therapies

Given the complexity of AD pathophysiology — and the growing consensus that amyloid buildup triggers tau spread at an early stage, with inflammation playing a critical role in toxicity of both — combination therapies are highly likely to be required in AD [63]. Within the context of gene and RNA-based therapies for AD and other dementias, at present therapies are specific to a single gene mutation (e.g., *GRN*) or proteinopathy (e.g., *MAPT*) to ensure clear evidence regarding safety and efficacy. Familial AD implicates hundreds of point mutations across *PSEN1, PSEN2*, and *APP* whilst sporadic AD involves amyloid and tau pathways. Whilst viral vector delivery of gene therapy at present has limited payload size, for intrathecal therapies several anti-amyloid and anti-tau RNA-based therapies could be combined within the same injection. Indeed, given the more direct action of these agents, there are fewer pharmacodynamic or pharmacokinetic barriers to combining agents within the same dose, especially for invasive procedures.

There is considerable debate over timing of delivery: not only what to give at what time during the preclinical and clinical disease course; but also should treatments be given sequentially or in parallel? Once disease processes have been controlled, what should maintenance therapy look like? As we develop genetic therapies, when and how should they be given in combination with other modes of action such as anti-amyloid immunotherapy and potentially immune-modulating therapy? To some extent the latter may depend on pragmatics, as anti-amyloid immunotherapies become standard of care in some regions. The answers are as yet uncertain, and will require testing in trial programs to ensure characterization of the safety and benefit of drug combinations before their utilization in clinical practice. However, in the future, combination therapies aiming to prevent and/or reduce amyloid and tau pathologies, and other treatment approaches, are envisaged.

### 3.13 Timing of intervention

There are multiple considerations for all stakeholders (patients, clinicians, and regulators) when deciding on timing of treatments. In principle, earlier treatment is preferred for any neurodegenerative disease to maximize quality of life. For inherited disease with known highly penetrant pathogenic mutations, treatment of pre-symptomatic disease cases to delay onset of clinical disease is a realistic aim in ADAD, particularly with increasing knowledge of the likely age of onset. Trials with amyloid immunotherapy treatments in pre-symptomatic disease are suggestive [64] and further work is ongoing (e.g., DIAN-TU Tau Next Generation, NCT05269394). In sporadic AD prediction of onset is less certain and so treatments have been generally administered to those with symptoms. As our ability to predict progression and prognosis improves this situation may change.

Genetic therapies are in early stages of development and have also been limited to symptomatic patients due to the as yet uncharacterized risks of these approaches. Early phase clinical trials in patients with early clinical AD usually focus on safety, tolerability, pharmacokinetics, and target engagement and disease activity biomarkers. This is especially important with the potential of “one and done” gene modification therapies for specific familial AD and/or amyloid pathways in general [30]. However, early stage clinical trials in symptomatic patients present problems when assessing efficacy, as pathology may be too advanced to be modifiable or reversable. As such, it is important to have good preclinical models of advanced disease to ensure human trials have a plausible chance of efficacy at later disease stages. Additionally, later disease stages may be confounded with other patient comorbidities. Once safety is better understood, clinical trial determinations of effectiveness can include participants with earlier symptomatic (e.g., PROCLAIM) or pre-symptomatic disease.

Furthermore, differing genetic therapies may be required based on putative AD phenotypes. For example, dementia in younger (75 years of age) adults is more strongly associated to hippocampal tau pathology than older (95 years of age) adults [65]. Additionally, in patients with early clinical AD with age below 75 years, a predominant amyloid phenotype is most evident in male *APOEɛ4* homozygotes, whereas female *APOEɛ4* noncarriers demonstrate a predominantly tau phenotype [[66], [67], [68]]. Such preliminary findings need further validation and characterization to determine the optimal interventional timing and effectiveness of amyloid- and tau-directed genetic therapies.

### 3.14 Treatment monitoring

From a drug development perspective, genetic therapies require clear markers of both target engagement and biomarker and/or clinical markers of efficacy. This allows clear and rapid decision-making in determining dose, dosing interval and efficacy to streamline feasibility assessments and ultimately reduce already high genetic drug development costs [69]. Moreover, this may provide early indications of populations likely to benefit based on either disease mechanism (e.g., autosomal dominant vs sporadic) or disease stage across the spectrum of disease severity. Beyond drug development, subsequent regulatory approvals may also depend on disease activity biomarkers [70].

Biomarkers of target engagement and efficacy typically include proximal and/or distal translational proteins (e.g., soluble APP and Aβ42 for mivelsiran, tau and ptau181 for IONIS MAPT_Rx_/BIIB080). Importantly though, markers of target engagement do not always translate into clinical benefits. For example, polyGP was reduced by the respective ASOs in the WVE-004 and BIIB078 trials in patients with ALS due to *C9orf72* hexanucleotide repeat expansions, and mutant huntingtin protein was reduced by the ASO tominersen in patients with HD_,_ but neither was associated with clinical benefits [58,71]. Potential causes may be pathogenic mechanisms not addressed (such as the antisense RNA strand in *C9orf72* ALS) [41,42], or intervention too late in the disease process. Additionally for RNA-based therapies, although mRNA levels and protein levels are correlated at steady-state, at times of stress or transition (potentially from healthy to disease with added neuroinflammatory changes) then these correlations may be perturbed and non-linear [72]. Other biomarkers require further understanding as to clinical relevance. For example, neurofilament light increased with tominersen [57] and a had subacute spike with PR006 [9], and ventricular volume increased with tominersen [58] and BIIB080 [2], all without clear clinical correlates. Moreover, in clinical practice, there are multiple potential confounders to determining biomarker levels across patients. Even with monogenic inherited AD, which should have a relatively uniform pathway, there may be several disease modifiers, such as *APOE4* and *SORL1* [73,74]. Thus, like for conventional trials, there is a need for genetic trials to recruit diverse populations to ensure potential confounds to biomarkers are widely captured and distributed.

Finally, given the extended duration of effects of genetic therapies from even a single dose, monitoring for long-term outcomes is particularly important. Long-term follow-up of various durations is recommended dependent on genetic therapy mechanisms [75]. Generally speaking, AAV therapies (typically for gene supplementation) recommend a minimum 5 year follow-up period, whereas gene editing approaches for 15 years. More detailed considerations are dependent on risk of latency and/or additional safety concerns from preclinical studies. The establishment and use of extended registries of patients receiving genetic treatments (not just gene editing) should be considered.

### 3.15 Dosing and duration

A key aim of present genetic therapies in Alzheimer’s disease is to reduce abnormal protein synthesis. However, the specific amount and duration of lowering required remains uncertain and may depend on several factors including disease stage and phenotype. Robust phase 2 studies for dose finding are encouraged, learning from trials such as the phase 1 and phase 3 studies of tominersen [57,58], and the potential for dose-dependent adverse effects of DRG toxicity in AAV therapies [54].

There is uncertainty about the duration of therapy for both conventional (i.e., small molecule and monoclonal antibodies) and genetic therapies, however, this is more of an issue for genetic therapies given the larger magnitude of change and much longer duration of effect. This may be of increased concern for treatments which target a normal pathway (e.g., amyloid and tau) since the long-term consequences of interference in these pathways remains unknown. The possible long term requirement for treatment, potentially before symptom onset and lasting decades, in particular those with genetic forms of disease such as ADAD or DS, raises concern for the effects of long-term perturbations to normal amyloid pathways. However, normalization of APP expression in DS may restore homeostasis with little concern as to long term loss of function of amyloidogenic pathways. Although preclinical studies can support the safety of therapies targeting normal pathways (e.g., BIIB080), detailed studies of protein turnover (e.g., via stable isotope labelling kinetics such as NCT06372821) can provide additional information.

### 3.16 Improved clinical to preclinical communication

Translation from preclinical science to clinical trials has a well-established pathway, however the inverse process also requires facilitation. Insights from clinical trials into drug-induced adverse effects, particularly class effects such as CSF pleocytosis and DRG toxicity, require further assessment of mechanisms, pathogenicity, and potentially mitigation and/or treatment. This can be facilitated by wider dissemination of trial results and adverse event data to the broader scientific community, to allow recruitment of academic, industry, and combined resources. Additionally, detailed publication of negative trial results can allow careful examination of the cause of negative trial findings and potential alternative mechanisms to advance drug development. For example, WVE-004 and subsequent sense and anti-sense ASO candidate strategies as discussed in frontotemporal dementia earlier. Additionally, review of the tominersen trial indicates possible failures due to inappropriate dose selection, high disease burden in participants, or off-target effects [76]. Such analysis is crucial as learnings can be applied more broadly to inform preclinical development and early phase trials of other genetic therapies.

Although this is not a specific problem to genetic therapies (e.g., the exact pathophysiology of ARIA remains unclear), given there is less experience and familiarity with adverse effect profiles of genetic therapies, ongoing specific research using animal models and long-term follow up of patients is to be encouraged. A model of circular translation from bench to bedside to bench would strengthen translational science in understanding these mechanisms.

### 3.17 Patient involvement

Patient involvement in the design and communication of genetic trials is crucial, with specific considerations for ADAD. Many patients are highly informed about developments in Alzheimer’s disease therapy and so may already be aware of genetic therapies and the implications. Other patients may need more education as genetic therapies may be more unfamiliar [77]. It is important to have an evidence-based way of communicating the mechanisms, benefits, and risks of genetic therapies to patients, study partners, and friends and family members.

Participant input into the design of clinical trials for genetic therapies is also paramount. Currently administered routes are invasive (predominantly intrathecal or intracisternal) with sometimes repeated CSF sampling to determine biomarkers of target engagement and treatment response. This needs discussion with patients regarding tolerability of recurrent invasive procedures, particularly balanced against relatively infrequent dosing. This is likely to change in the future for some drugs with the development of active transport mechanisms.

Furthermore, when discussing trial design and implementation with all stakeholders, accurate patient and study partner input into risk/benefit analysis is crucial. Several considerations must be taken into account, such as whether patients are asymptomatic (and in the case of ADAD, how long until symptom onset), or if already symptomatic then the degree of symptoms and the risk/benefit considerations for first-in-human studies. This needs to be balanced against perceived discomforts of medication administration, as well as concerns about unknown side effects and the irreversibility of treatments. Patient engagement is needed in genetic trial design to ensure that appetite for risk is not under- or over-estimated. Lessons learnt from current trial design, such as including patients and family members on steering committee in DIAN-TU, should be adapted to genetic trials.

## 4 Conclusion

Genetic therapies for Alzheimer’s disease and other dementias hold promise. Specific advantages include targeting of all forms of a particular protein, regardless of compartment location; extended durations of therapeutic action – ‘one and done’ in the case of gene therapies; very infrequent dosing in RNA therapies;; potential to radically alter the course of disease. Despite these opportunities, there are unique basic science and clinical challenges in target validation, drug delivery, optimal disease-stage timing for intervention, and implementation in clinical practice. While genetic therapies are in the early stages of development, development can be accelerated by developing biological subclassifications of AD and other dementias, leveraging lessons learned in safety and efficacy in earlier studies of genetic therapies, and from their application in other medical conditions. Combined insights from academia and industry can work together to develop effective treatments for symptomatic disease, and potentially for prevention in those with pre-symptomatic disease.

## CRediT authorship contribution statement

**D. Jakabek:** Writing – review & editing, Writing – original draft. **A.M. Isaacs:** Writing – review & editing, Conceptualization. **B. De Strooper:** Writing – review & editing, Writing – original draft, Conceptualization. **M. Tuszynski:** Writing – review & editing, Writing – original draft, Conceptualization. **R. Lane:** Writing – review & editing, Writing – original draft, Conceptualization. **O. Uspenskaya:** Writing – review & editing, Writing – original draft, Conceptualization. **E. McDade:** Writing – review & editing, Writing – original draft, Conceptualization. **M.S. Rafii:** Writing – review & editing, Writing – original draft, Conceptualization. **C.J. Mummery:** Writing – review & editing, Writing – original draft, Conceptualization.

## Conflicts of Interest

The authors declare that they have no known competing financial interests or personal relationships that could have appeared to influence the work reported in this paper.
